# Morphological improvement after multi-directional cranial distraction osteogenesis procedure for syndromic craniosynostosis

**DOI:** 10.3171/2021.1.FOCVID20116

**Published:** 2021-04-01

**Authors:** Masahiro Kameda, Eijiro Tokuyama, Takaya Senoo, Isao Date

**Affiliations:** Departments of 1 Neurological Surgery and; 2 Plastic and Reconstructive Surgery, Okayama University Graduate School of Medicine, Dentistry and Pharmaceutical Sciences, Okayama, Japan

**Keywords:** craniosynostosis, morphological improvement after multidirectional cranial distraction osteogenesis, Apert syndrome, midsagittal vector analysis

## Abstract

The multidirectional cranial distraction osteogenesis (MCDO) procedure, which uses an external distraction device, enables tailor-made distraction in an arbitrary direction, eliminating the disadvantage of unidirectional distraction with an internal distraction device. Multiple-suture synostosis cases for syndromic craniosynostosis patients are better indicated for this procedure. Here the authors describe seven cases in which the MCDO procedure was used to treat syndromic craniosynostosis. In each case, the MCDO procedure and postoperative distraction, with reference to midsagittal vector analysis of normal morphology in Japanese children, resulted in morphological improvement.

The video can be found here: https://vimeo.com/519006555

## Transcript

**0:22 Overview of Case**. Here we demonstrate MCDO procedure and postoperative distraction for a 3-year-old Apert syndrome boy who had a past history of tracheostomy for subglottic stenosis.

**0:32 Preparation Before Surgery**. Before surgery, we analyzed his skull shape with the reference of midsagittal vector analysis of normal morphology of the Japanese children. The finding was sticking out forehead and small volume in the posterior part of the skull. We performed preoperative model surgery using patient particular 3D skull actual size model. Then, we prepared intraoperative surgical guides made from dental impression silicon, which would be used for osteotomy navigation. We sterilized and brought it in the surgery.^1,2^

**1:10 Surgery and Demonstration of Skin Incision**. Under general anesthesia, he was placed in the prone position. We placed bicoronal skin incision and performed subgaleal dissection of the scalp to expose skull. The temporal muscle was attached with the scalp flap. To design the actual osteotomy lines, the surgical guide was put on the skull.

**1:35 Placement of Anchor Pin of the MCDO Flame and Drilling in the Posterior Part of the Skull**. Three or four screw holes for the anchor pins were drilled in the temporal bones under the guide hole of the template. The traction pins were fixed in the center of the occipital bone. According to this particular surgical guide, the skull was osteotomized with diamond drill and craniotome.

**2:33 Change Position and Drilling in the Anterior Part of the Skull**. After closing the wound, he was placed in the supine position. Opening the wound again, we performed subgaleal dissection of the scalp was followed by subperiosteal dissection from 10 to 15 mm above the supraorbital rim. According to his particular surgical guide, the skull was osteotomized with diamond drill into small pieces. The individual bone pieces were not peeled off from the dura in order to preserve vascularity of bone pieces. We also performed fronto-orbital advancement using a reciprocating saw. After reshaping, supraorbital bar was returned and fixed with absorbable plates. For future distraction, two traction pins were fixed in the supraorbital bar. Several bone pieces were connected with absorbable plates in the operation in order to distract these pieces in one lump.

**3:51 Closure of Incision and Fixing MCDO Frame**. After closing the wound, the traction pins and anchor pins were penetrated the scalp. In this case, we used the T-type MCDO frame, which covers not only anterior part of the skull but also posterior part of the skull, whereas standard type of MCDO frame covers only anterior part of the skull. We fixed MCDO frame with the anchor pins in the temporal bones. The wires secured in the traction pin holes were passed through the holes in the MCDO frame and connected to the distractor, so that the bone pieces could be pulled in the appropriate direction. Finally, the wires were fixed to the distractors attached on the MCDO rame.

**4:46 Postoperative Distraction and Postoperative Management**. Approximately 1 week after operation, we started postoperative distraction 1–2 mm per day. In order to get better shape of skull, we performed distraction according to the normal morphology of Japanese Children based on the midsagittal vector analysis.^3^ As a result of midsagittal vector analysis, his skull was bigger compared with average of 3-year-old Japanese children, which was why we performed distraction with the reference average + 1 standard deviation of 4-year-old Japanese children.

At the final phase of distraction period, we tweaked distraction length by checking CT images. Patient had 1-month consolidation period, followed by removal of MCDO frame. His shape of the skull was dramatically changed by MCDO.

We have performed MCDO procedure for seven syndromic craniosynostosis cases. In each case, MCDO procedure and postoperative distraction with the reference of midsagittal vector analysis for normal morphology of Japanese children result in morphological improvement.

**6:08 References** ^1–5^

## Author Contributions

Primary surgeon (neurosurgery): Kameda. Primary surgeon (plastic and reconstructive surgery): Tokuyama, Senoo. Editing and drafting the video and abstract: Kameda. Critically revising the work: Kameda. Reviewed submitted version of the work: all authors. Approved the final version of the work on behalf of all authors: Kameda. Supervision: Date.
